# The choice of response alternatives in COVID-19 social science surveys

**DOI:** 10.1371/journal.pone.0263552

**Published:** 2022-11-23

**Authors:** Daniel B. Wright, Sarah M. Wolff, Rusi Jaspal, Julie Barnett, Glynis M. Breakwell

**Affiliations:** 1 Department of Educational Psychology and Higher Education, University of Nevada, Las Vegas, NV, United States of America; 2 Vice Chancellor’s Office, University of Brighton, Brighton, United Kingdom; 3 Psychology Department, University of Bath, Bath, United Kingdom; 4 Institute of Global Health Innovation, Imperial College, London, United Kingdom; Coventry University, UNITED KINGDOM

## Abstract

Social science research is key for understanding and for predicting compliance with COVID-19 guidelines, and this research relies on survey data. While much focus is on the survey question stems, less is on the response alternatives presented that both constrain responses and convey information about the assumed expectations of the survey designers. The focus here is on the choice of response alternatives for the types of behavioral frequency questions used in many COVID-19 and other health surveys. We examine issues with two types of response alternatives. The first are vague quantifiers, like “rarely” and “frequently.” Using data from 30 countries from the Imperial COVID data hub, we show that the interpretation of these vague quantifiers (and their translations) depends on the norms in that country. If the mean amount of hand washing in a country is high, it is likely “frequently” corresponds to a higher numeric value for hand washing than if the mean in the country is low. The second type are sets of numeric alternatives and they can also be problematic. Using a US survey, respondents were randomly allocated to receive either response alternatives where most of the scale corresponds to low frequencies or where most of the scale corresponds to high frequencies. Those given the low frequency set provided lower estimates of the health behaviors. The choice of response alternatives for behavioral frequency questions can affect the estimates of health behaviors. How the response alternatives mold the responses should be taken into account for epidemiological modeling. We conclude with some recommendations for response alternatives for behavioral frequency questions in surveys.

## Introduction

People’s reactions to mask and COVID-19 vaccine regulations lay bare the need for social science research to complement biological and economic research for effective management of epidemics. The underlying data for this research often come from responses to sample surveys. Constructing survey questions that lead to valid, reliable, and fair responses is difficult [1]. In everyday conversations, people ask questions without constraining others to respond using pre-defined formats with limited options, but in survey conversations this often occurs. This is done to ease the coding of responses and often to get the respondents to translate their complex beliefs into a single value that is more suitable for statistical analyses.

The responses from health surveys are critical for monitoring health related behaviors, evaluating health campaigns, understanding/modeling the spread of disease, and developing public policy [2]. Health behavior data inform resource allocation decisions and provide necessary information to identify target groups for intervention, to track progress, and to evaluate existing strategies [3]. The current research was prompted by the COVID-19 epidemic and the realization that one of the main reasons for its level of impact in many countries is people failing to heed guidelines from scientific groups on the efficacy of health related behaviors (e.g., hand washing, mask wearing, vaccines). Epidemiologists use estimates from surveys to gauge how much people follow these guidelines.

Self-reports are prone to bias and memory errors [4]. In order to understand measurement error within surveys, researchers examine the cognitive processes that occur when respondents answer questions [5–9]. The theoretical approach taken here is to assume the survey situation is an artificial conversation, and like other conversations respondents use the information presented to them to interpret the meaning of questions [10]. For modeling epidemiology, the focus is often on estimates from behavioral frequency questions [11], like “how often do you wash your hands?” Our focus is on the response alternatives. As these are not part of most everyday conversations, when they are presented in surveys they may stand out. When responding to a survey question, the responses are often ordered, and respondents will see this as a scale composed of words. According to Grice’s maxims, [12, 13] when people are presented with a question they assume that the information–including that within the prescribed response format–will be accurate and relevant. While the survey is an artificial conversation, respondents may still assume that the scale and words are appropriate for the behavior in the question [10].

Respondents assume that researchers construct a meaningful scale that reflects appropriate knowledge about the distribution of the behavior. Accordingly, values in the middle range of the scale are assumed to reflect the ‘average’ or ‘typical’ behavior, whereas the extremes of the scale are assumed to correspond to the extremes of the distribution.

Schwarz (2010, p.49) [14]

Respondents go through several steps while answering behavioral frequency questions. According to Schwarz & Oyserman [15] these are:

1. Understanding the question
2. Recall relevant behavior
3. Inference and estimation
4. Map the answer onto the response format
5. Editing the answer for reasons of social desirability

While described as steps, information at each step can inform previous steps. For example, at step 4 the respondent may use the response scale to inform how to understand the question and to define the behaviors. The scale could affect what the respondents think about the behavior in the question stem. If a person is unsure of population norms and is presented with a set of response alternatives suggesting the behavior is common, they may come to believe that the behavior is common. Another possibility is that the response alternatives could change what the respondents think the target event is. Wright and colleagues [16] describe how this can occur for vague and ambiguous terms. They asked respondents how often their teeth were cleaned either with response alternatives suggesting this meant by a dentist or with response alternatives suggesting this meant by themselves. Which set respondents received affected what respondents thought the target behavior was. This also occurs for vague behaviors like being annoyed or being satisfied. [17, 18] For relatively well defined events (e.g., how many cups of coffee you have in a typical day), the definitions should not be greatly affected. If the difference between the population norms implied by the response alternatives and their pre-survey beliefs is great, this might cause them to doubt the applicability of the scale, or to change their normative beliefs. Finally, respondents can interpret any set of alternatives as a scale from low to high, ignoring the particular words used to compose the scale. This will be more likely when the response alternatives are vague. In these cases it is unclear what question they answer: how much they engage in the behavior compared with others; with their expectations; with their behavior before the pandemic; etc. This is discussed further at the end of the paper when we make recommendations for the choice of response alternatives.

The response alternatives for behavioral frequency questions can have many different formats. This includes: free recall (how many times have you done this behavior in a time period), when was the last time you did the behavior, how often do you do the behavior compared with others, filling out a diary or a calendar, using vague quantifiers, and sets of numeric alternatives. The focus of this paper is on the final two of these formats.

## Study 1: Vague quantifiers

Schaeffer titled her paper “Hardly ever or constantly” as reference to a scene in the film *Annie Hall* where Alvie Singer and Annie Hall are each ask how often they sleep together [19]:

Alvie: Hardly ever, maybe three times a week.
Annie: Constantly, I’d say three times a week.

This highlights that vague quantifiers can be associated with different numeric values for different people. This can create issues for comparing groups.

Part of the differences in how people use vague quantifiers can be attributed to what they think the normative behaviors of that behavior are. Wright and colleagues [20] asked UK respondents how much they thought people typical watched television, and found large differences in this belief by social class. In a subsequent study they asked the following two questions.


$$\begin{array}{lll} \text{Q1} & \text{And}\;\text{from}\;\text{this}\;\text{list,}\;\text{on}\;\text{average,}\;\text{how}\;\text{much}\;\text{television}\;\text{do} & \text{None}\;\text{at}\;\text{all}\\ & \text{you}\;\text{watch}\;\text{on}\;\text{a}\;\text{typical}\;\text{weekday?} & \text{Hardly}\;\text{any}\\ & & \text{A}\;\text{little}\\ & & \text{Quite}\;\text{a}\;\text{bit}\\ & & \text{A}\;\text{lot}\\ \text{Q2} & \text{And}\;\text{about}\;\text{how}\;\text{many}\;\text{hours}\;\text{would}\;\text{that}\;\text{be?} & \text{[Free}\;\text{Recall]} \end{array}$$

They found that respondents from classes that watched more television and gave higher mean responses for each vague quantifier than did respondents from classes that watched less television. Groups that thought people tended to watch more television interpreted the vague quantifiers as corresponding to higher amounts. Using a large multi-country database, we examine the association between responses using vague quantifiers and a free recall numeric response. Both of these attempt to measure the frequency of the behavior.

Rather than social class, the current study estimates the amount each country does the target behavior and examines if this is a good indicator of how people in the country interpret the vague quantifiers. This study uses data from thirty countries and the survey was delivered in many languages. The surveys used different languages, as appropriate, and as such the specific vague quantifiers used may have different meanings. Our prediction is that countries where there is a higher mean for the numeric response, will have higher numeric values corresponding to the different vague quantifiers (and their translations). We split the data into 25% for estimating the means for the behaviors and then used the remaining 75% to test the prediction.

### Methods

Data from https://github.com/YouGov-Data/covid-19-tracker [21] were downloaded on November 8, 2021. All their data are anonymized. The database and discussion of their methods are available at www.coviddatahub.com. In total, at the time of download, there were 734,075 respondents. There are four survey questions of interest. The first variable is the country. There are thirty countries. Sample sizes of those with complete responses are shown in Table 1. The next three variables of interest are two vague quantifier questions and one free recall question about hand-washing/sanitizing. These were separated by 17 questions. Their wording, from the UK version (i.e., *sanitising*), is shown in Table 2. Only data where there were no missing values for these were used, leaving 646,177 cases.

**Table 1:** Countries in the imperial database, number of respondents with complete data for the questions analyzed here, and the mean for the natural logarithm of the responses to the free recall question, plus a starting value of +.5, for the 25% of the training sample (given the sample sizes these are very similar to the total means).

Country	n	mean(ln(x+.5))	Country	n	mean(ln(x+.5))
Australia	35,932	2.03	Brazil	10,308	2.26
Canada	31,060	2.15	China	15,985	1.30
Denmark	32,154	2.39	Emirates	11,924	2.16
Finland	19,076	2.07	France	36,360	2.07
Germany	36,271	1.93	Hong Kong	7,042	1.77
India	16,145	2.07	Indonesia	12,141	1.90
Israel	6,480	1.88	Italy	36,106	2.19
Japan	16,931	1.49	Malaysia	12,133	1.91
Mexico	12,012	2.31	Netherlands	11,399	1.93
Norway	32,032	2.21	Philippines	12,002	2.07
Saudi Arabia	11,450	1.95	Singapore	32,977	1.84
South Korea	15,644	1.72	Spain	36,198	2.06
Sweden	36,201	2.11	Taiwan	11,993	1.53
Thailand	12,085	1.76	UK	46,143	2.16
US	27,919	2.02	Vietnam	12,074	1.75

**Table 2:** Vague quantifier and free recall hand washing/santizing questions from the Imperial data set.

Question	Resp. Alts.
Washed hands with soap and water	Always Frequently Sometimes Rarely Not at all
Used hand sanitiser	Always Frequently Sometimes Rarely Not at all
Thinking about yesterday … about how many times, would you say you washed your hands with soap or used hand sanitiser?	[Free recall]
Variable names are as found in the Imperial webpage.	

### Results and discussion

The vague quantifier questions are not directly comparable to the free recall question as the latter combines the two behaviors asked about in the vague quantifier questions. We begin by comparing responses from each of vague quantifier questions with the free recall responses question. A small percentage of respondents (0.07%) gave responses of 1,000 or more to the free recall question. Assuming these respondents are awake for 18 hours, 1,000 is approximately once a minute. This is a very skewed variable (skew = 25.06). It was transformed using *ln*(*x* +.5) (the +.5 as some people, 1.22%, said zero) and this lessened the skewness to 0.14.

**Fig 1:**
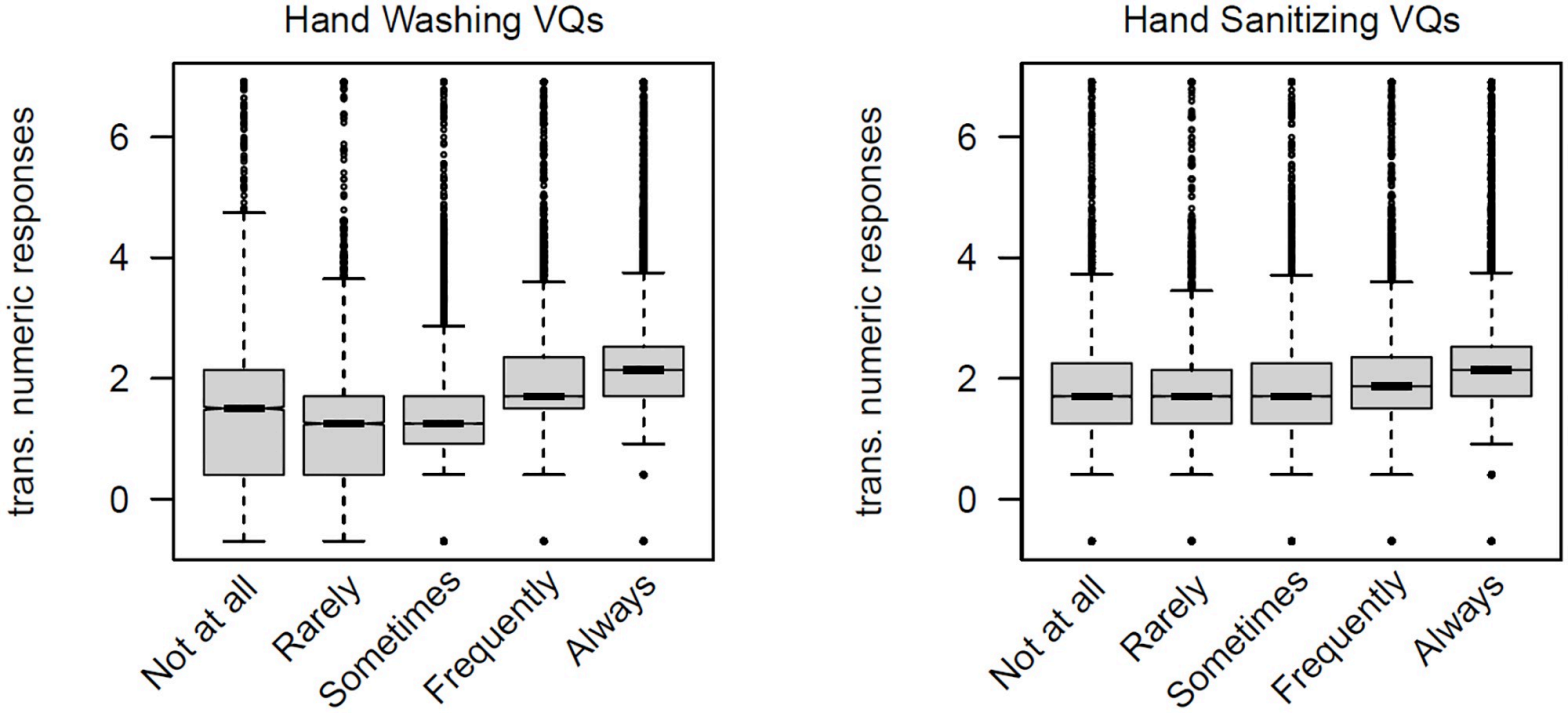
Boxplots for the transformed numeric response from the free recall question for the two vague quantifier questions.

The associations between the vague quantifier responses and the transformed numeric responses from the free recall question are shown in Fig 1. The relationship between these two variables are fairly weak, even allowing for them asking about slightly different behaviors. Twenty-five percent of the data were used to estimate the mean for each country for the transformed (*ln*(*x* +.5)) responses from the free recall question. These are shown in Table 1. The remaining 75% of the data are used to explore the relationship between the transformed numeric responses and the vague quantifiers. Given the large sample size, the 25% and 75% are sufficient for our purposes. Since there are separate vague quantifier questions for hand washing and hand sanitizing, these will be examined separately.

**Fig 2:**
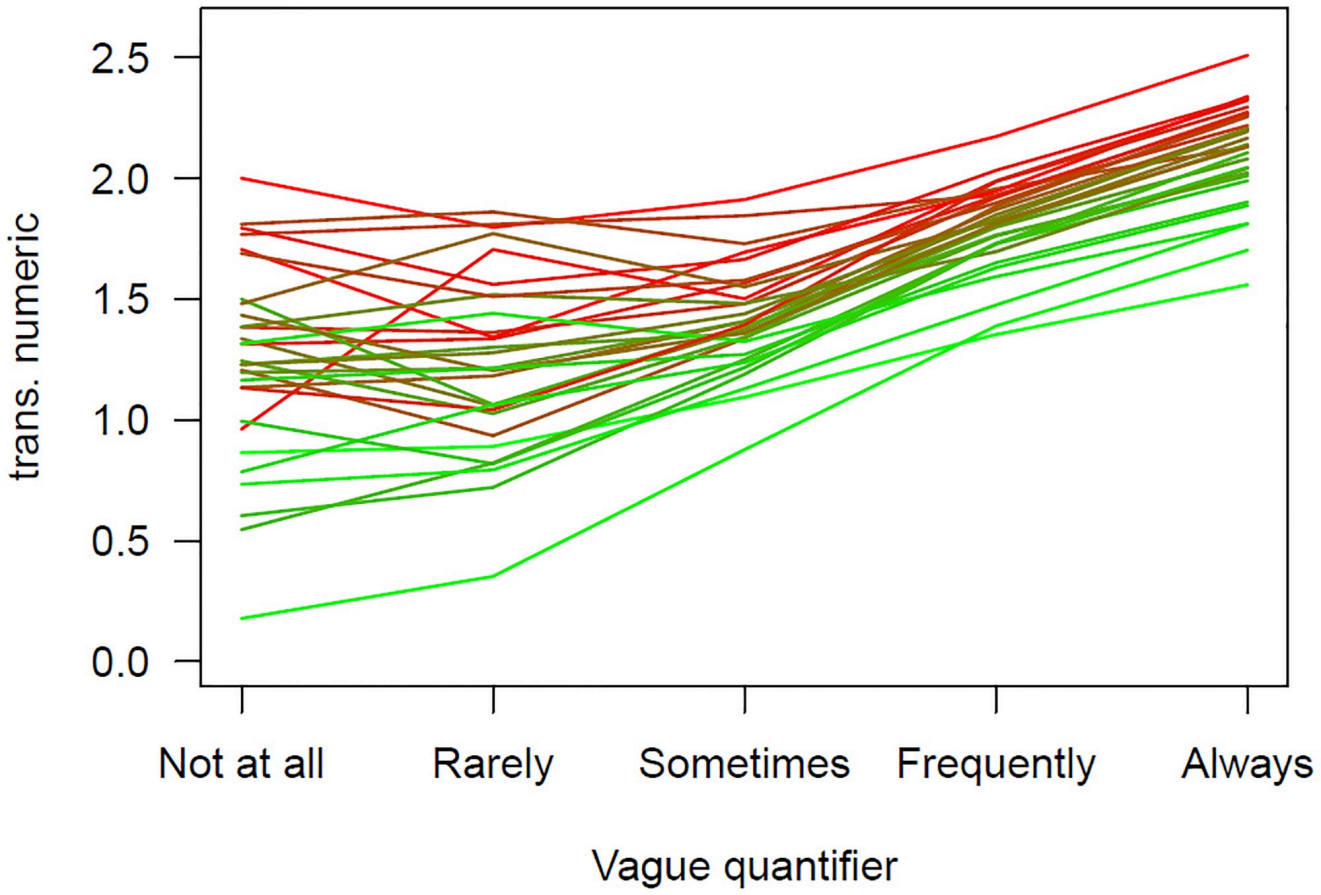
The relationship between the means of the transformed free recall responses for each vague quantifier by country for the vague quantifier washing question. Countries with low free recall responses from the 25% of the sample are shown in green and those with high values in red, with intermediary countries shown in a mixture of these two colors.

The hand washing vague quantifier variable is treated as categorical, so with *df* = 4 for its five categories. When it is used to predict the transformed variable from the free recall question, the *R*^2^ value was.119. Including the categorical variable of country, with its *df* = 29, increased this to.177. The critical question is how much of this difference, Δ*R*^2^ =.058, can be accounted for by the single variable (df = 1) corresponding to the mean of the transformed free recall variable taken from the other 25% of the total sample? If there was 1/29th increase the value would be about 3% of this amount, so approximately:.121. Including this single df variable the *R*^2^ =.175, or 96.38% of the possible amount. Fig 2 shows this. The color corresponds to the mean for the transformed free recall responses. The greener the line the lower the country mean for the transformed values (the color is based on a gradient between green and red, so countries with means near the middle appear brown-ish). As is clear, the greener lines are lower than the redder lines.

**Fig 3:**
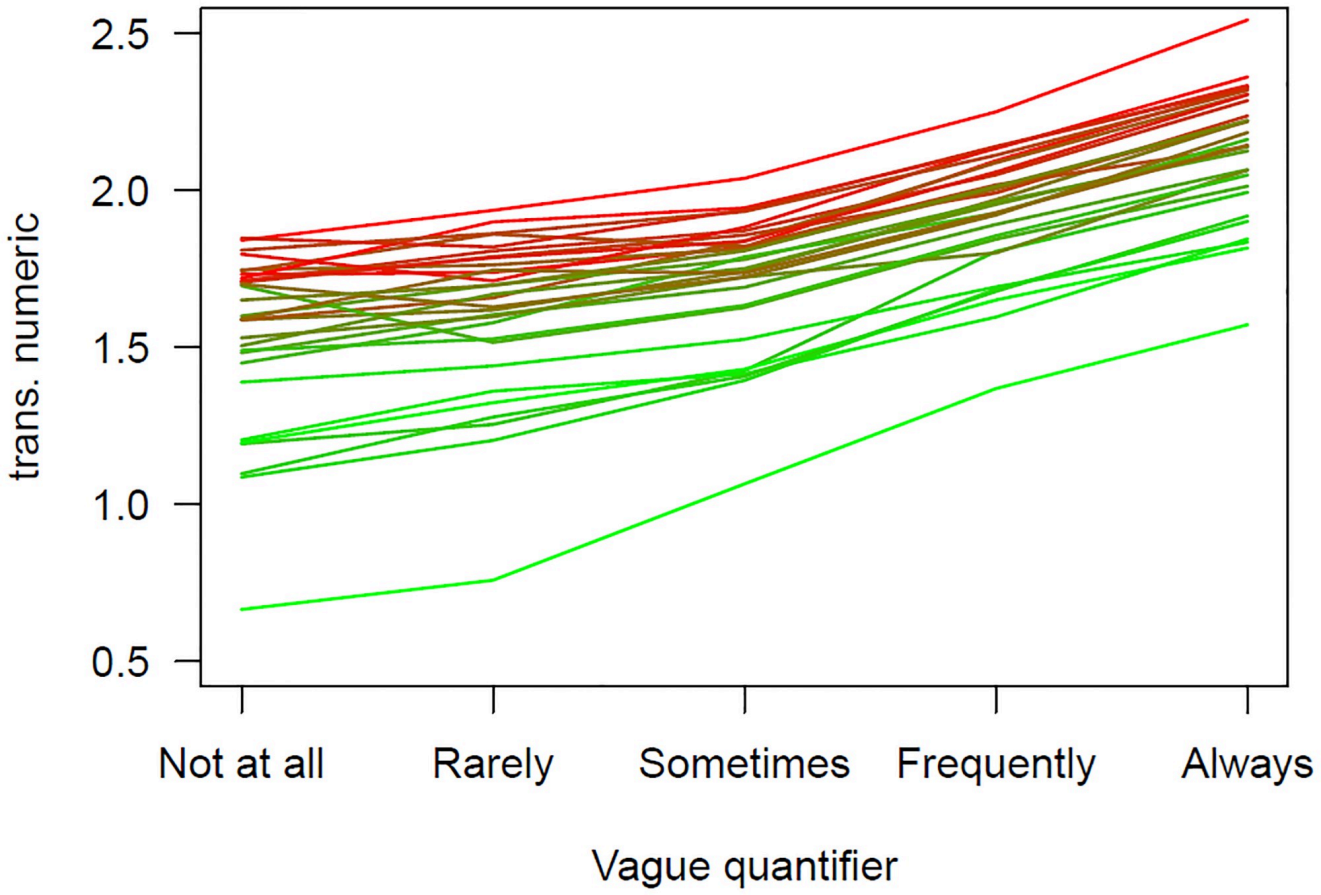
The relationship between the means of the transformed numeric estimates for each vague quantifier by country for the vague quantifier hand sanitizer question. Countries with low free recall responses from the 25% of the sample are shown in green and those with high values in red, with intermediary countries shown in a mixture of these two colors.

The findings were similar comparing the hand santizing vague quantifier variable with the transformed free recall responses. When just the vague quantifier variable is used to predict these, the *R*^2^ value was.081. Including the categorical variable of country increased this to.143, and difference of Δ*R*^2^ =.058. Using the single country mean variable produced an *R*^2^ =.140, or 93.97% of the possible amount (as opposed to 3%). This is shown in Fig 3 with the greener lines being below the redder lines.

There are two main conclusions from this study:

1. Vague quantifiers mean different things to different people and this makes it problematic to compare results from these questions.
2. Some of the differences in the ways in which people use vague quantifiers is accounted for by the variation in behaviors among the person’s linguistic community.

## Study 2: Numeric response alternatives

The purpose of the COVID-19 Household Pulse Survey was to examine the effects of the pandemic on variables ranging from mental health behaviors to vaccine perceptions [22]. The data are meant to help government direct aid, assistance, and support to the people and places that need it most. An example question reads:


$$\begin{array}{ll} \text{Over}\;\text{the}\;\text{last}\;7\;\text{days,}\;\text{how}\;\text{often}\;\text{have}\;\text{you}\;\text{been}\;\text{both-} & \text{Not}\;\text{at}\;\text{all}\\ \text{ered}\;\text{by}\;\text{the}\;\text{following}\;\text{problems}\;.\;.\;.\;\text{Feeling}\;\text{nervous,} & \text{Several}\;\text{days}\\ \text{anxious,}\;\text{or}\;\text{on}\;\text{edge?}\;\text{Would}\;\text{you}\;\text{say}\;\text{not}\;\text{at}\;\text{all,}\;\text{several} & \text{More}\;\text{than}\;\text{half}\;\text{the}\;\text{days}\\ \text{days,}\;\text{more}\;\text{than}\;\text{half}\;\text{the}\;\text{days,}\;\text{or}\;\text{nearly}\;\text{every}\;\text{day?} & \text{Nearly}\;\text{every}\;\text{day}\\ \text{Select}\;\text{only}\;\text{one}\;\text{answer} & \end{array}$$

Schwarz and colleagues [10, 18, 23–25] conducted a series of studies to show how the choice of which response alternatives to present can affect behavior estimates. Schwarz applies Grice’s maxims of communication [12] to the survey situation to explain his findings. Consider the survey question above. The question asks respondents to reflect over the last several days. Three of the four response alternatives involve the event happening multiple days in the previous week: several days; more than half the days; and nearly every day. According to Schwarz this may make respondents feel that being nervous, anxious, or on edge, are likely to occur with greater frequency in the population than if the response alternatives were: more than once a month, once a month, and none.

The behaviors used by Schwarz and colleagues were specifically chosen to show the methodological effects predicted by his hypotheses. The behaviors used here were chosen because of their relation to disease transmission and that they are part of health guidelines (e.g., those from the Center for Disease Control and Prevention [CDC] in the US, and the National Health Service [NHS] in the UK). Also, Schwarz et al. used in person (face-to-face), pen-and-paper, and telephone administration modes. Nowadays, online surveys are becoming more common so the current study uses the online administration mode. Our primary research question is whether the choice of response alternatives affects the estimates of several health related behaviors. Respondents are randomly allocated into one of two conditions. Those in the first condition are asked behavioral frequency questions with response alternatives that discriminate more finely at low frequencies (low frequency condition) and those in the second condition are asked these questions with response alternatives that discriminate more finely at high frequencies (the high frequency condition). The expectation from past research is that those in the low frequency condition will provide estimates that equate with lower values for the behaviors than those in the high frequency condition.

### Methods

The study involved the creation of new data using human subjects. It received IRB approval from the UNLV IRB [1753484-2].

#### Sample

Respondents were recruited via Amazon Mechanical Turk (MTurk). All respondents were over 18 years old. The provided written informed consent on MTurk before progressing to the survey.

The MTurk system allows what are called MTurk workers to sign up to participate in different tasks, most of which offer some compensation. The researcher can create the task, here a survey using Qualtrics, and MTurk directs them to the task and provides a way to compensate the workers (MTurk also receives a fee). To be an MTurk worker you need to be 18 or over and with US social security number. Additional requirements to sign up for this study were that respondents had to have 100 previous what are called MTurk human intelligence tasks (HITs) and to have at least a 95% satisfaction rating from those conducting the research. Respondents were compensated $2 for completing the questionnaire.

A *catcha* question was included in the Qualtrics survey to help ensure respondents were paying attention. Not correctly completing this meant the survey would not be included, but all passed this (or did not complete the survey). There were two additional exclusion criteria: multiple uses of an IP address and responding too quickly. While each MTurk worker needs a separate US social security number, people could use multiple MTurk accounts. A more likely reason is that people from within the same household are responding using the same IP address. As these people may talk about the study before the second completes it (and would in other ways also be non-independent), the duplicates (i.e., not the first one using the IP address) were excluded. Respondents who on average responded faster than two seconds for the behavioral frequency and attitude questions were also excluded. The number of people excluded for these reasons, in both conditions, is shown in Table 3.

**Table 3:** Respondent flow showing the allocation into conditions and those excluded for a duplicate IP addresses or responding too quickly.

	Number of Respondents: Total n = 695	
	Low Response Alternatives	High Response Alternatives
Total Recruited	345	350
Duplicate IP	16	19
Too Quick	9	10
Total excluded	25	29
Percentage excluded	7%	8%
Final analysed (total n = 641)	320	321

#### Materials and experimental design

Respondents were asked three behavior/health questions related to COVID-19:

- How often did you wash your hands?
- When you washed your hands, typically how long did you spend?
- In a typical day, how often did you apply hand sanitizer?

Respondents were randomly assigned to have either the low or the high condition as listed in Table 4.

**Table 4:** The response alternatives for the low and the high frequency conditions. The dashed lines show how the raw data can be re-coded for comparable frequencies/durations.

Question	Low	High
Hand washing frequency	Never	3 or less
	1	
	2–3	
	4–6	4–6
	7 or more	7–10
		11–20
		More than twenty
Hand wash duration	less than 2–3 seconds	5 seconds or less
	5 seconds	
	10 seconds	10 seconds
	20 seconds	20 seconds
	More than 20 seconds	One minute
		More than one minute
Hand sanitiser	Never	3 or less
	1	
	2–3	
	4–6	4–6
	7 or more	7–10
		11–20
		More than twenty

Respondents were then asked seven attitude questions shown below. Respondents used a 0–100 sliding scale. Responses were measured to the tenth, for example 29.4. Our analyses concerning these variables are exploratory and concern whether the set of response alternatives that were presented affects the responses on these variables. Being in the low condition may make respondents think they perform those behaviors more than most, and being in the high condition may make respondents thing they perform those behaviors less than most. The first two items below explore if this also impacts the relative concerns people express toward economic and health impacts. We have no predictions for the direction of effects for the remaining five.

- Compared with other people, how much were you concerned with the economic impacts of the pandemic?
- Compared with other people, how much were you concerned with the health impacts of the pandemic?
- Thinking back to the previous twelve months, how concerned were you about catching COVID-19 yourself?
- Thinking back to the previous twelve months, how concerned were you about yours friends and family catching COVID-19?
- Suppose that you were supposed to meet a small group of people. If you were not feeling well (slight fever, running nose), how likely would you have stayed at home?
- Suppose that you were not feeling well (slight fever, running nose), how likely is it that you would have consulted a medical professional?
- People vary in how much they trust scientists with respect to many issues. Please rate your view.

In addition, respondents were asked their year of birth, gender, ethnicity, and asked to rate their political beliefs on a 0–100 liberalism/conservativism scale.

The data were collected on a Monday evening, May 17, 2021, and this was a few days after President Biden relayed CDC advice that masks need not be worn by fully vaccinated people indoors in the USA (later, with the increased spread of new variants, guidance changed).

#### Statistical plan

The behavioral frequency questions are treated in two ways. When treating them as 1–5 rating scales, the means of these three are compared between the two groups using Hotelling’s *T*^2^. When the responses are re-coded into matching semantic categories (within the dashed lines of Table 4), they are treated as categorical variables and compared using *χ*^2^ tests, Cramér’s *V*s, and multinomial logistic regressions. The attitude questions are compared using *t*-tests, and the *p*-values adjusted using Holm’s procedure. The pre-registration states that the associations among the responses to these seven questions would be examined and if they correlated highly a single analysis would be done. In the original submission this was what we report. The new analysis based on a reviewer’s suggestion is appropriate because the first two items involve comparison and thus would be more likely to be affected. We agree that this is the more appropriate approach. The conclusions reached are the same. The difference in means for that single factor were non-significant.

### Results and discussion

Three related sets of statistical analyses are conducted for examining the behavioral frequency estimates in this study. The first examines if there are differences in responses for the two groups if the response labels are not considered. As such, the questions are all treated as 1–5 rating scales for this set of analyses. The null hypothesis of equal means for the two groups would be true if respondents did not use the response labels when answering these questions. The second set of analyses involves re-coding responses into matching categories, as shown in Table 4. Here the null hypothesis corresponds to respondents not being influenced by whether the response alternatives differentiate more at low or at high frequencies. This is important for epidemiology because if these values differ it would produce different estimates for the frequencies of these behaviors. The third set of analyses is exploratory, investigating if there are carryover effects from which set of response alternatives were presented to a series of attitude questions.

#### Do the verbal labels affect responding?

Table 5 shows the group means when treating the data as 1–5 rating scales, the differences in means, effect sizes for these differences (Cohen’s *d*), and the 95% confidence intervals for these effect sizes. The values of Cohen’s *d* are from 0.65 to 1.11. Cohen describes 0.5 as a *medium* effect and 0.8 as a *large* effect [26]. Generic verbal labels for effect sizes can be problematic because the absolute meaning of any effect size is context dependent [27, 28]. Here they are used to compare the relative size of these effects with those reported below when the response variables are treated as categorical.

**Table 5:** Descriptive statistics for the raw values, 1–5, for the three behavior answers. This coding ignores the verbal labels, so these differences show some people pay attention to the labels.

	${\bar{x}}_{low}$	${\bar{x}}_{high}$	diff	LB	UB	*d*	*LB*_*d*_	*UB*_*d*_
Hand wash frequency	4.20	3.10	-1.10	0.95	1.25	1.11	0.94	1.27
Hand wash duration	3.82	3.19	-0.63	0.50	0.76	0.75	0.59	0.91
Hand sanitizer frequency	3.22	2.24	-0.98	0.78	1.17	0.75	0.59	0.91

Hotelling’s *T*^2^ [29] is used to test if, as a group, the means of these three variables differed by condition. Box’s M, [30] which tests equality of covariances, was statistically significant result, *χ*^2^(6) = 60.38, *p* <.001. Therefore a version of Hotelling’s test that allows for heterogeneity of covariance matrices was used, and (as expected from the effect sizes) it was statistically significant: *F*(3, 619.52) = 236.15, *p* <.001. Thus, the respondents did use the labels when constructing their response.

#### Do the response alternatives affect behavioral frequency estimates?

The second set of analyses examines the re-coded responses within the dashed lines of Table 4. Table 6 shows the proportions for each of these categories for the two conditions. Cramér’s *V* (with bias correction) is used here as the effect size measure. The BCa bootstrap intervals (2,000 replications) are shown. The *χ*^2^ value is for testing the null hypothesis, *V* = 0. Cohen [31] provides verbal labels for these effect sizes, and they vary by the degrees of freedom. For *df* = 2 (three categories), these are: small =.071, medium =.212, and large =.354. For *df* = 3 (four categories), these are: small =.058, medium =.173, and large =.289. As a group, using Cohen’s terminology, these show medium to large sized effects. Which set of response alternatives were presented did affect the behavioral frequency estimates.

**Table 6:** Statistics comparing the recoded responses for the three behavior questions by condition.

Question	Cond.	Category				*V* (95% CI)		*χ*^2^(*df*) (all *p* <.001)
		1	2	3	4			
Hand wash frequency	Low	.20	.38	.42		*V* =.27	(.19,.34)	*χ*^2^(2) = 47.78
	High	.07	.25	.68				
Hand wash duration	Low	.06	.30	.41	.24	*V* =.22	(.13,.28)	*χ*^2^(3) = 31.85
	High	.01	.16	.53	.30			
Hand sanitizer	Low	.57	.26	.17		*V* =.22	(.14,.29)	*χ*^2^(2) = 31.82
	High	.40	.22	.37				

#### Are there carryover effects to a set of attitude questions?

The final question addressed for this study is whether the set of response alternatives that were used had carryover effects to a set of attitude questions. Because those given the response alternatives focused on high frequencies may have felt they performed these events less than most people, in comparison to those given those focused on the low frequencies, we felt this might have carryover effects when asked how concerned they are, compared with others, about the economic and health issues related to COVID. We include several other questions to gauge if any carryover effect extend to other COVID beliefs. Table 7 shows the means for all seven questions for both conditions, along with the difference in means, and test statistics. As there are seven tests, the Holm adjusted *p* values are reported, and none of these were statistical significant. Further, the most likely items to show carryover effects are the first two items. Neither of these had statistically significant un-adjusted *p*-values. Thus, we do not find sufficient evidence to conclude that there are carryover effects here.

**Table 7:** Statistics comparing the responses on the attitude questions for those given the response alternative focused on low values and those on high values. The *t* and *p* values used the normed data (i.e., ranked and the inverse normal function applied) due to distributions being skewed.

	${\bar{x}}_{low}$	${\bar{x}}_{high}$	diff	LB	UB	*t*	p	*p*_*adj*_	*d*
Compared: economic	6.42	6.48	0.06	-0.45	0.34	-0.35	.724	1.000	-0.02
Compared: health	6.88	6.87	-0.01	-0.39	0.41	-0.31	.756	1.000	0.00
You catching COVID	6.47	6.30	-0.18	-0.30	0.66	0.68	.499	1.000	0.06
Friends catching	7.36	7.16	-0.21	-0.23	0.64	1.38	.169	1.000	0.07
If ill, stay home	8.57	8.62	0.04	-0.40	0.31	0.24	.813	1.000	-0.02
If ill, consult Dr.	6.13	6.04	-0.09	-0.40	0.57	0.34	.737	1.000	0.03
Trust scientists	8.16	7.86	-0.30	-0.03	0.63	2.45	.015	.102	0.14

## Discussion and recommendations

Evaluating public health campaigns and modeling disease spread requires estimating people’s behaviors. This is usually done using surveys. Traditionally much effort has focused on the wording of question stems, and less on the response alternatives. Two studies focus on the response alternatives. The first shows that comparing responses across countries using vague quantifiers is difficult, but for these data differences among the countries can almost completely be accounted for by the norms in that country. While this stresses a difficulty making these comparisons, because the country-differences can be accounted for by another variable, a simple theory, following [20], can be posited:

The meanings of the vague quantifiers are partially based on what respondents believe the survey designers believe about the population norms. Much of the respondents’ beliefs will be based on the norms of their social and national groups.

There will be specific linguistic nuances of particular words and geographic variation in their usage. Further research would be necessary to construct improved ways, based on these differences, to estimate numeric values. However, as noted in the recommendation section, vague quantifiers should not be used if want to estimate or to compare groups with respect to numeric values.

The second study was a randomized experiment to measure the effect of using different response alternatives to estimate the frequency of health related behaviors. The results showed that when several of the response alternatives were for high frequencies, respondents gave answers corresponding to higher estimates than when they were for low frequencies. The choice of response alternatives should be carefully considered when estimating health related behaviors. Further, comparing estimates from surveys that use different sets of response alternatives should be done with great caution, if at all.

### Recommendations for response alternatives

Behavioral frequency questions require respondents to answer questions about past events. We make recommendations for two types of behaviors: rare and frequent. Whether an event type is rare or frequent will depend on the sample and there will be overlap between them, so survey designers should consider all the suggestions below where appropriate.

#### Rare events

Consider what is hopefully a rare event for the respondent, like a hospitalization, catching COVID-19, or being laid off. It is likely for most respondents these are rare and it is also likely that answers to these questions will be important both for the survey flow (e.g., on a COVID-19 survey if you answer YES to having COVID-19, you might be asked further questions), and the estimates for these will be important for epidemiological models. Therefore, accurate and precise answers are likely very important. The focus here is on the response alternatives, but it is important to consider the memory limitations, even for rare events (i.e., rare does not imply memorable), and that guiding the respondent using procedures like the cognitive interview, as the term is used in eyewitness research, should be considered [32]. In addition to remembering an event, respondents usually need to provide information about *when* the event occurred. People have difficulty saying when an event occurred [33], and this is where issues about the response format are most relevant.

Assuming that the respondents have time to respond to the survey and are near their cell phone, respondents should be encouraged to consult resources (e.g., emails, texts, vaccination card) to provide exact dates. If this information is not available, then respondents could be presented with an *event history calendar*, as devised by Belli and colleagues [34], and used in many health surveys [35]. These allow respondents to fill in notable events with known dates, like a child’s birthday and holidays, onto the calendars, and then allow the respondent to think where the event in question happened in relation to these. If using the event history calendar is impractical, most online surveys allow a calendar response so the respondent can give a precise date, and this can also be used if there were multiple incidences in the time frame. For long duration events a start and end could be provided, and respondents could also provide a range of dates if they are uncertain.

#### Frequent events

By frequent events we mean those that, for most respondents, are likely to occur multiple times each week (e.g., handwashing, eating a piece of fruit). As with rare events it is important to consider the cognitive limitations of the respondent, and in particular whether the respondent is likely to use some estimation heuristic or try to recall and count all episodes. Each of these has potential biases (e.g., people are more likely to not remember an event than to create a false memory for a non-existent event, e.g., [36], so recalling each incident is likely to result in an under-estimate), so if the accuracy is of much importance diary methods and experience-sampling methods [37] can be used, though these require coordination with respondents prior to the survey and in the case of experience-sampling methods more technology. For some frequent events, there might be available resources available retrospectively (e.g., filtering through the trash for some food consumption events), though these would not be available for most event types and would be more effort than is likely appropriate. The following assumes these resources are not available and that this is for a single retrospective survey.

Survey designers can be interested in both well-defined events and those that are not well-defined. While standard practice encourages survey questions to be well-defined, if the goal is to compare groups on, for example, how worried they are about catching COVID-19, or some other psychological state, there is often no way to make these precise. Because of this, it will be difficult to interpret numeric estimates of the frequencies with much confidence unless it can be made clear to respondents what, in this case, an episode of worry would be. In these situations it can be prudent to use vague quantifiers. The vagueness of the response alternative matches with the event. Often non-numeric responses can also be used. For example, comparison questions can also be used when this matches what people’s beliefs about the events are like. This would be a situation where pre-testing using think-aloud protocols would likely be valuable to show how respondents think about these event descriptions [38]. An alternative would be to construct a scale for “worry” whichever construct was appropriate, using several items. While the issues with response alternatives would still be present for each item, having multiple items would allow the researchers more psychometric tools to investigate the fairness, validity, and reliability of the measure [39].

For well-defined events, vague quantifiers should be avoided. The choice should be between free recall procedures and a set of numeric response alternatives. Free recall does not mean just writing something in a box. For online surveys, the software can force respondents to enter text in a specified format (e.g., as a number if that is necessary for subsequent analysis, rather than accepting, for example, a respondent writing “about 4 to 8, maybe”). An issue with this is that people’s memories may not be that precise. The granularity of people’s memories varies [40]. This can be accounted for when you ask for numeric information by allowing people to provide a range of values corresponding to their confidence [41]. Alternatively, sets of numeric response alternatives can be used, though this has the limitation that the alternatives will usually be a range of values so may not be as precise of free recall methods. However, study 2 showed the the choice of response alternatives can make a difference. Even a large set of response alternatives, it will still frame the response and provide information that the respondent may assume reflect population norms. However, a large set of alternatives is better for this aspect than a small set. Asking for some form of free recall response is often better [15]. However, it can be cognitively taxing and lead to differences in response strategy, for example using a heuristic to guess a frequency versus episodic enumeration.

## Summary

Survey data are used in social science research for informing economists about consumer behaviors, for health researchers evaluating compliance with different campaigns, for sociologists and psychologists constructing theories of why people behave in they ways that they do, and for many other purposes. Behavioral frequency questions have a special place within survey methods as researchers often translate responses into numeric estimates for the behaviors, and sometimes the precision of these estimates is critical. This is the case for epidemiologists predicting trends for the COVID-19 pandemic. While people often focus on the way the event itself is described in the question stem, there is less focus on the response alternatives. We focus on the response alternatives.

Our first study examined how people, across thirty countries, answered questions about hand washing and hand sanitizing. We found that people in different countries interpreted the vague quantifiers used as response alternatives differently. Most of the differences among countries could be accounted for using estimates of the behavior in the countries. We do not recommend using vague quantifiers with relatively well defined events like hand washing, but allowing people to provide numerical estimates. Our second study showed that care is still necessary when doing this. Using different sets of response alternatives produced different estimates of the behavior. One consequence of this is that comparisons between studies that use different sets of response alternatives should be done cautiously, if at all.

The choice of response alternatives should be carefully considered and the deciding how to construct them may be difficult. It may require careful pilot research and techniques like cognitive interviewing and in particular think-aloud protocols [38]. We provide a list of recommendations to allow researchers to start thinking about their choices of response alternatives for behavioral frequency questions.
